# Supplementary material for: The genome sequence of the brown tree ant, Lasius brunneus (Latreille, 1798) (Hymenoptera: Formicidae)
Source: Wellcome Open Res. 2026 Jun 24;11:372. [Version 1] doi: 10.12688/wellcomeopenres.26818.1 (PMC13555074; doi:10.12688/wellcomeopenres.26818.1)
Supplement: Peer review history

# Reviewer report: Reviewer response for version 1

**DOI:** 10.21956/wellcomeopenres.29529.r159235

**Published:** 2026-07-14

**Authors:** Leland Carl Graber

**Affiliations:** 1 American Museum of Natural History, New York, USA

In this article, Crowley and Telfer describe the process of sequencing and annotating the genome of *Lasius brunneus*. The methods in this article go into great detail about the process the authors took to prepare the specimen for sequencing, the genome assembly, and the post-assembly processing. All of the necessary data is shared through the article and through online repositories linked through the article.

 While this article is more than acceptable in its current form, I would recommend that the authors go into slightly more detail in the "Background" section. It might be helpful for the reader to understand more about the phylogeny of *Lasius brunneus*, and perhaps if other members of the genus or related genera have had their genomes sequenced in the past.

Are sufficient details of methods and materials provided to allow replication by others?

Yes

Is the rationale for creating the dataset(s) clearly described?

Partly

Are the datasets clearly presented in a useable and accessible format?

Yes

Are the protocols appropriate and is the work technically sound?

Yes

Reviewer Expertise:

I study insect systematics and phylogenetics, with a focus on ants and hemitperans.

I confirm that I have read this submission and believe that I have an appropriate level of expertise to confirm that it is of an acceptable scientific standard.

# Reviewer report: Reviewer response for version 1

**DOI:** 10.21956/wellcomeopenres.29529.r159226

**Published:** 2026-07-14

**Authors:** Adria C LeBoeuf

**Affiliations:** 1 University of Cambridge, Cambridge, UK

The molecular work, assembly, and annotation are of high quality and the manuscript is essentially ready. I have only a few minor suggestions that would improve the biological framing:

1. Please add a sentence on the ecological context of the sampled individuals. Were they from the same colony (presumably!). Given that *L. brunneus* is a tree-nesting species, noting the substrate (e.g., host tree species, whether the nest was in a living tree, a stump, or deadwood) would help readers and aid reproducibility.
2. I understand the DToL mission, but a sentence on why it is worth while having a genome for this species would strengthen the introduction. 
3. It would help to state whether the assembly is broadly consistent with other *Lasius* genomes in size and chromosome number, or whether anything is unusual. 

Are sufficient details of methods and materials provided to allow replication by others?

Yes

Is the rationale for creating the dataset(s) clearly described?

Partly

Are the datasets clearly presented in a useable and accessible format?

Yes

Are the protocols appropriate and is the work technically sound?

Yes

Reviewer Expertise:

Social insects, omics, evolutionary biology, behaviour

I confirm that I have read this submission and believe that I have an appropriate level of expertise to confirm that it is of an acceptable scientific standard.

# Reviewer report: Reviewer response for version 1

**DOI:** 10.21956/wellcomeopenres.29529.r159229

**Published:** 2026-07-13

**Authors:** Ralph S Peters

**Affiliations:** 1 Leibniz Institute for the Analysis of Biodiversity Change, Bonn, Germany

The submitted data note accompanying indexation of the genome assembly of Lasius brunneus is a fine contribution, outlining the results and underlying methods in state-of-the-art way.

 The species selected is a common widespread ant species, and the genome will be a highly welcome resource. I suggest adding a few words to the background section on the economic importance of the species and/or its status as pest species. This is not really relevant in Britain, but for other parts of Europe. One or two references on this will be sufficient; it will also add to the relevance and visibility of the note and the genome because many will know L. brunneus as a pest species.

Are sufficient details of methods and materials provided to allow replication by others?

Yes

Is the rationale for creating the dataset(s) clearly described?

Partly

Are the datasets clearly presented in a useable and accessible format?

Yes

Are the protocols appropriate and is the work technically sound?

Yes

Reviewer Expertise:

Evolution, biodiversity discovery, Hymenoptera

I confirm that I have read this submission and believe that I have an appropriate level of expertise to confirm that it is of an acceptable scientific standard.
